# Efficient Targeted Mutagenesis Mediated by CRISPR-Cas12a Ribonucleoprotein Complexes in Maize

**DOI:** 10.3389/fgeed.2021.670529

**Published:** 2021-05-12

**Authors:** Shujie Dong, Yinping Lucy Qin, Christopher A. Vakulskas, Michael A. Collingwood, Mariam Marand, Stephen Rigoulot, Ling Zhu, Yaping Jiang, Weining Gu, Chunyang Fan, Anna Mangum, Zhongying Chen, Michele Yarnall, Heng Zhong, Sivamani Elumalai, Liang Shi, Qiudeng Que

**Affiliations:** ^1^Syngenta Crop Protection, Research Triangle Park, Durham, NC, United States; ^2^Integrated DNA Technologies, Coralville, IA, United States

**Keywords:** CRISPR-Cas12a, AsCas12a, LbCas12a, mutant Cas12a, ribonucleoprotein delivery, maize genome editing

## Abstract

Recent advances in the development of CRISPR-Cas genome editing technologies have made it possible to perform targeted mutagenesis and precise gene replacement in crop plants. CRISPR-Cas9 and CRISPR-Cas12a are two main types of widely used genome editing systems. However, when CRISPR-Cas12a editing machinery is expressed from a transgene, some chromosomal targets encountered low editing frequency in important crops like maize and soybean. Here, we report efficient methods to directly generate genome edited lines by delivering Cas12a-gRNA ribonucleoprotein complex (RNP) to immature maize embryos through particle bombardment in an elite maize variety. Genome edited lines were obtained at ~7% frequency without any selection during regeneration *via* biolistic delivery of Cas12a RNP into immature embryos. Strikingly, the gene editing rate was increased to 60% on average and up to 100% in some experiments when the Cas12a RNP was co-delivered with a PMI selectable marker gene cassette and the induced callus cultures were selected with mannose. We also show that use of higher activity Cas12a mutants resulted in improved editing efficiency in more recalcitrant target sequence. The advances described here provide useful tools for genetic improvement of maize.

## Introduction

Clustered Regularly Interspaced Short Palindromic Repeats (CRISPR)/CRISPR-associated (Cas)- adaptive immune systems are widely distributed in nature to defend bacteria from invasion of phages and other mobile genetic elements, such as plasmids and transposons (Sapranauskas et al., 2011; Hille et al., 2018). CRISPR-Cas systems are classified into two main classes and six types (Bayat et al., 2018). The most widely used CRISPR-Cas systems for genome editing are the Types II and V members of the CRISPR/Cas Class 2 systems, Cas9 and Cas12a (aka Cpf1) effectors, respectively (Tang and Fu, 2018). CRISPR/Cas9 system has 2 functioning parts, the Cas9 endonuclease and a guide RNA (gRNA) comprised of two components, a target specific CRISPR RNA (crRNA) and a trans-activating RNA (tracrRNA) (Jinek et al., 2012; Memi et al., 2018). In contrast to Cas9, Cas12a (Cpf1) has several distinct features such as T-rich protospacer-adjacent motif (PAM), a guide crRNA without the need for trans-activating RNA (tracrRNA), creation of sticky ends, and ability to self-process crRNA in addition to the DNA nuclease activity (Zetsche et al., 2015; Bayat et al., 2018). These RNA-guided Cas nucleases (RGNs) scan the genome to search for target DNA sequences complementary to the gRNA and generate a DNA double-strand break at the target sequence if there is a proper protospacer adjacent motif (PAM) present. The resulting chromosomal break is repaired by the host DNA repair machineries, either through non-homologous end joining (NHEJ) or homology-directed repair (HDR) (Chakrabarti et al., 2019).

CRISPR-Cas systems are flexible, precise, simple to use, and efficient. Both CRISPR-Cas9 and CRISPR-Cas12a nucleases have been used widely as highly sequence-specific tools for efficient genome modification (Murugan et al., 2017). CRISPR-Cas systems also have enormous potential for improving global crop production. Their uses in editing genomes have been expanding rapidly among different crops. CRISPR/Cas9 system has been used for genome editing in *Arabidopsis*, tobacco, rice, sorghum, maize, wheat, poplar, tomato, soybean, petunia, sweet orange as well as liverwort *Malcantia polymorpha* (El-Mounadi et al., 2020). Several engineered Cas9 enzymes with altered fidelity or target recognition specificity and Cas9 proteins fused with other types of DNA modification enzymes have also been developed (Sretenovic et al., 2020; Zhang Y. et al., 2020). For example, SpCas9-NGv1, an engineered CRISPR-Cas9 recognizing NG PAM edits has been shown to efficiently edit endogenous target sites with NG PAMs in both rice and *Arabidopsis*; furthermore, target-specific base editors have been generated by fusing NGv1 nickase to cytidine deaminase (Endo et al., 2019).

CRISPR-Cas12a is an alternative system to CRISPR-Cas9 for genome editing (Zetsche et al., 2015). The functionality of Cas12a systems for genome editing has been reported in many plant species including *Arabidopsis*, cotton, rice, tobacco, tomato and maize (Endo et al., 2016; Begemann et al., 2017; Tang et al., 2017; Xu et al., 2017; Zhong Z. et al., 2018; Lee et al., 2019; Li et al., 2020). LbCas12a variants, targeting alternative non-canonical PAMs, have also been used to edit plant genome sequences and broadened the range of targetable sequences by Cas12a (Li et al., 2018a). Recently, multiplex gene editing with CRISPR-Cas12a and CRISPR-Cas9 systems has also been achieved by expressing the nuclease and crRNA array from a single Pol II promoter for plant genome editing (Wang et al., 2018). Besides, synthesis-dependent repair of Cas12a-induced double strand DNA breaks enabled targeted gene replacement in rice (Li et al., 2018b). However, the editing efficiency is usually lower with Cas12a than with Cas9 and the efficiency varies considerably among different maize targets (Lee et al., 2019). It is possible that temperature is one of the main factors affecting Cas12a editing efficiency (Malzahn et al., 2019). Recently, a high activity variant of Cas12a with temperature tolerance was also reported to resulting in improved editing efficiency in plants (Schindele and Puchta, 2020).

Other than the activity of CRISPR-Cas system, efficient delivery of the editing machinery into the plant cells is key to genome editing applications in crops. In general, stable transformation of DNA expression vectors encoding the CRISPR-Cas components is achieved using *Agrobacterium tumefaciens*-mediated delivery or direct delivery method such as particle bombardment and protoplast transformation. However, stable transformation of plants is a long process and generates transgenic plants with CRISPR-Cas expression cassettes integrated into their genomes. The integration of DNA construct encoding for the editing machinery in plant genome and its continuous expression might lead to unwanted modifications at off-target genomic sequences (Murovec et al., 2018). Delivering ribonucleoproteins (RNPs) or RNA into cells without DNA is an alternative method to generate transgene-free targeted genome edits (Woo et al., 2015; Svitashev et al., 2016; Zhang et al., 2016; Andersson et al., 2018; Liang et al., 2018). DNA-free delivery is also preferred in crops that are vegetatively propagated since it is not an option to use crossing to breed out the transgene inserts (Murovec et al., 2018; Que et al., 2019). Genome editing through DNA-free delivery with RNP may also ease up regulatory concerns related to transgenes. Editing through transfection of protoplasts with pre-assembled Cas9 RNP complexes has been demonstrated in many plant species (Woo et al., 2015; Malnoy et al., 2016; Svitashev et al., 2016; Andersson et al., 2018; Liang et al., 2018; Murovec et al., 2018; Sant'Ana et al., 2020). However, due to the technical difficulties and long timeline in regenerating plants from protoplasts, the routine use of protoplasts with RNP delivery for generating of stably edited lines has not been reported in economically important crops including cotton, maize, soybean and wheat. On the other hand, some of these crops have well-established high efficiency biolistic transformation systems and successful generation of heritable edits has been achieved using CRISPR-Cas9 *in vitro* transcripts or RNPs delivered with biolistic method (Svitashev et al., 2016; Liang et al., 2018).

During our research in applying Cas12a editing system to maize we have also observed low editing efficiency in some editing targets when the Cas12a editing machinery was expressed from integrated transgenes. In order to understand why we observed variable editing efficiencies, we have tested several parameters such as different Cas12a enzymes and delivery methods. Here we report efficient gene editing in maize with Cas12a RNP delivered into leaf protoplasts and immature embryos. When RNP was delivered via particle bombardment, the average editing efficiency in immature embryos is above 60% using co-selection with the phosphomannose isomerase (PMI) marker gene. A large proportion of mutants generated using this protocol carried biallelic mutations, suggesting efficient cleavage of target sites by Cas12a RNP. The results also indicate that Cas12a RNP-mediated editing has much reduced target dependency. We also show that edited plants can be generated with DNA-free RNP delivery method without any selection in a shorter timeline, albeit at much lower efficiency. We also compared different versions of AsCas12a and LbCas12a genes and showed that higher activity Cas12a mutants resulted in improved editing efficiency at recalcitrant target sequences.

## Materials and Methods

### Plant Materials

Syngenta's proprietary elite maize inbred variety NP2222 was used in all experiments. Stock plant care, maize ear production, immature embryo extraction and transformation with PMI as selectable marker gene was carried out as described before (Zhong H. et al., 2018).

### Cas12a and crRNA Reagents Used for Plant Transformation

Cas12a (aka. Cpf1) enzymes including AsCas12a-WT, AsCas12a-V3, AsCas12a-Ultra and LbCas12a-V3 (Supplementary Table 1) (Behlke et al., 2018; Vakulskas et al., 2020; Zhang L. et al., 2020) and crRNA were purchased from or provided by IDT (Integrated DNA Technologies, Inc. USA). Guide or spacer sequence of the crRNAs and their corresponding maize gene target are shown in Supplementary Table 2. The crRNA scaffold used for LbCas12a is based on CRISPR-LbCpf1 system, while the crRNA scaffold used for AsCas12a is based on CRISPR-AsCpf1 system (Zetsche et al., 2015).

### CRISPR-Cas12a Expression Vectors and Generation of Transgenic Maize Plants

Binary transformation vector 12672 contains 2 gene expression cassettes, one for the PMI selectable marker gene driven by the maize Ubiquitin-1 promoter and another for the AmCyan fluorescent protein gene driven by the maize Ubiquitin-1 promoter (Zhong H. et al., 2018). For expression of LbCas12a, the rice codon-optimized coding sequence (Tang et al., 2017) was used with 3 bp changes to remove 2 Bsp119I and one RsrII recognition sites. The Cas12a transformation vectors (Supplementary Table 3, Supplementary Figure 1) contain 3 expression cassettes: Cas12a expression cassette driven by sugarcane ubiquitin-4 promoter (prSoUbi4) followed by the NOS terminator, crRNA-ribozymes expression cassette and PMI selectable marker cassette. The *Bx9* gene target sequences for the crRNAs of these Cas12a vectors are listed in Supplementary Table 3. Transgenic plants were generated through particle bombardment using isolated immature embryos as targets and PMI as a selectable marker (Wright et al., 2001; Chen et al., 2018; Zhong H. et al., 2018). Briefly, immature embryos were isolated from harvested ears at about 9–11 days after pollination and pre-cultured for 1–3 days on osmoticum media. Pre-cultured embryos were then bombarded with the DNA described above using the BioRad PDS-1000 He™ Biolistic particle delivery system. Bombarded embryos were then incubated in callus induction media. For 33°C treatment, bombarded embryos were incubated at 33°C for 2 days before moving back to regular culture temperature at 28°C. Induced calli were then moved onto mannose selection media. Mannose resistant calli were transferred to regeneration media to induce shoot formation. Shoots were then sub-cultured onto rooting media. Leaf samples were harvested from rooted plants for Taqman® assays to detect mutations in the target site using a previously described real time quantitative polymerase chain reaction (qPCR) Taqman^®^ method (Ingham et al., 2001; Chen et al., 2018).

### RNP (Ribonucleoprotein) Complex Preparation, Particle Bombardment, and Plant Regeneration

To generate Cas12a-crRNA RNP complexes, 0.3 nmol of Cas12 protein and 0.3 nmol of crRNA were mixed together in a total volume of 11 μl and incubated at room temperature for 10 min. For RNP delivery alone, the RNPs were coated onto 0.6 μm gold particles (Bio-Rad, USA) as follows: 100 μl of gold particles (water suspension of 10 mg/ml) and 20 μl of glycogen (20 mg/ml) were added to the premixed RNPs, mixed gently, and then incubated on ice for 10 min. For co-delivery of RNP with DNA, the RNPs and DNA vector plasmid 12672 were coated onto gold particles as follows: 100 μl of gold particles (water suspension of 10 mg/ml) and 20 μl of glycogen (20 mg/ml) were added to the premixed RNPs and DNA vector, mixed gently, and incubated on ice for 10 min. The RNP/DNA coated gold particles were then centrifuged at 8,000 g for 40 s and the supernatant was removed. The pellet was resuspended with 30 μl of sterile water by brief sonication, and then spread onto a macrocarrier disc (10 μl each) followed by air dry in the laminar flow hood (2–4 h). Immature embryos were isolated, pre-cultured and bombarded with RNP complex alone or RNP complex with 12672 plasmid DNA as described above for DNA delivery. For experiments with RNP delivery alone and those that did not go through mannose selection, the embryos were cultured on callus induction medium for 2 weeks before transferring to regeneration medium for shoot regeneration. Shoots were then sub-cultured onto rooting media. For RNP-DNA delivery, induced calli were selected on mannose containing media and the resistant calli were placed on regeneration media for transgenic plant production as described above for generation of transgenic plants containing Cas12a expression vectors.

### Etiolated Maize Leaf Protoplast Isolation and Transfection

Protoplasts were isolated from etiolated maize leaves grown under dark conditions as described (Sheen, 1991). Cas12a RNP was assembled as above. Protoplast transfection was carried out as described, with modification (Sant'Ana et al., 2020). Transfection reactions consisted of 5 x 10^5^ protoplasts per reaction and were incubated with PEG solution (40% PEG-4000, 0.6 M Mannitol, 100 mM CaCl_2_) for 15 min. Following termination by W5 solution (154 mM NaCl, 125 mM CaCl2, 5 mM KCl and 2 mM MES, pH 5.7), transfected protoplasts were resuspended in 300 μl W1 solution (0.6 M Mannitol, 4 mM MES, pH 5.7, 4 mM KCl), transferred to 96-well clear bottom microplate and incubated for 2 days in the dark at 28°C without shaking. DNA was isolated from transfected protoplasts after 2 days and analyzed for mutant identification (Chen et al., 2018).

### Molecular Analysis and Mutant Identification

For detection of mutation in immature embryos bombarded with Cas12a-crRNA RNP complex, genomic DNA was extracted from embryos cultured for 2 days after bombardment. Genomic sequences flanking the crRNA target sites were PCR-amplified by specific primers: Bx9-Forward (5′- AAACA CTAAA CACTC CCCTC TG−3′), Bx9-Reverse (5′- GTTTA CCCAT CTCTT TTAAC ACTAT−3′), MIR604-Forward (5′- GGATA TGACT CCACT GACCA−3′) and MIR604-Reverse (5′- CATTT CTCCA TAGCC CGTTT−3′). Amplicons were purified through Ampure XP beads (Beckman Coulter Inc., Brea, CA) following the manufacturer's instructions. Samples containing 40 μl of > 1 ng/μl of purified amplicon DNA were sequenced by SeqWell Inc. (Beverly, MA). NGS raw FASTQ files were aligned to corresponding reference sequences. Reads with Indels were extracted. The reads were further filtered by two criteria: first, the variant region must overlap with gRNA target sequence and second, the variant size must be equal to or larger than 2 bp. The percentage of reads with edits was calculated using the number of filtered Indels divided by total read count covering the gRNA target region. For analysis of editing in regenerated plants, leaf samples were harvested, and total genomic DNA was extracted from rooted plants for Taqman assays were used to detect putative mutations in target sites (Ingham et al., 2001, Chen et al., 2018). Identified putative mutants were further characterized by Sanger or NGS sequencing analysis.

## Results and Discussion

### Editing of Chromosomal Target Sequences by Cas12a RNP Delivered Into Maize Immature Embryos and Leaf Protoplasts

DNA-free delivery of editing reagents in the form of protein, mRNA or RNP has great potential to address potential regulatory requirements and public concerns associated with the incorporation of recombinant DNA molecule into edited plants. Successful genome editing using CRISPR-Cas9 RNPs delivered using the biolistic method has been previously demonstrated in wheat and maize using immature embryos as explants (Svitashev et al., 2016, Liang et al., 2018). However, biolistic delivery for Cas12a RNP has yet to be established in maize. We were therefore interested in investigating whether Cas12a RNP could be delivered similarly using particle bombardment to achieve efficient editing in maize. We chose two genomic regions as targets: one is the genic region of *Bx9* gene which encodes for the UDP-glucosyltransferase involved in benzoxazinoid DIMBOA biosynthesis (von Rad et al., 2001) (GRMZM2G161335 in B73 RefGen_v3) (Sequence File 1 in Supplementary Material) and another is the non-genic region corresponding to the transgene insertion locus of a commercial event MIR604 for root worm control (Chen et al., 2018), (MIR604FS, Sequence File 2 in Supplementary Material).

Table 1 shows the transient editing results of target sequences by AsCas12a-Ultra RNP complexes delivered via particle bombardment. The results show that some genomic targets such as Bx9TS2 can be edited efficiently using AsCas12a RNP. Since only a small percentage of the embryo cells (i.e., those at the scutellum surface and adjacent 1–2 cell layers) can be targeted by microparticles during bombardment, it is expected that a maximum of 5–10% of the total cells in an immature embryo explant can receive Cas12a RNP. Assuming a 10-day immature maize embryo has around 1,000 cells, it has a maximum of 50–100 cells that can be targeted by the delivered RNP complex. Assuming an editing efficiency of ~50%, it is estimated that only 25–50 cells will get edited and this translates into 2.5–5.0% of sequence reads having mutant variants. It is remarkable that one of the tested crRNAs (crBx9GS2) resulted in 3.42% reads with edits, suggesting that AsCas12a RNP has been delivered to many of the surface cells and it is very active in editing the *Bx9* gene sequence. Other tested crRNAs resulted in lower editing efficiency, with crMIR604GS2 and crMIR604GS4 having 0.35 and 0.19% edited reads, and crMIR604GS1 and crMIR604GS3 having the lowest percentage of edited reads (~0.10%) (Table 1). The reason for lower editing efficiency is not clear, but it is possible both gRNA and target accessibility may play a role. The results suggest that RNP delivery directly into immature embryos, the transformation target explants, can be a quick way to screen gRNAs for Cas12a-mediated editing.

**Table 1:** **Transient editing of maize chromosomal target sequence by AsCas12a-Ultra RNP complexes delivered via particle bombardment of immature embryos.**.

**Experiment ID**	**Target site**	**crRNA**	**Total sequence read number**	**Variant (InDel) read number**	**% Reads with edits (Mean ± SD)** **(rep number)**
Blank control 1	MIR604TS2	None	12,841	1	0.01% (*n =* 1)
Blank control 2	MIR604TS4	None	14,410	2	0.01% (*n =* 1)
MIR604T1A	MIR604TS1	crMIR604GS1	39,267	38	0.10% ± 0.10% (*n =* 3)
MIR604T2A	MIR604TS2	crMIR604GS2	37,750	133	0.35% ± 0.24% (*n =* 3)
MIR604T3A	MIR604TS3	crMIR604GS3	15,922	15	0.09% ± 0.04% (*n =* 3)
MIR604T4A	MIR604TS4	crMIR604GS4	36,445	69	0.19% ± 0.18% (*n =* 3)
Bx9T2A	Bx9TS2	crBx9GS2	35,040	1,198	3.42% ± 0.18% (*n =* 3)

We also validated the top performing crRNAs (crBx9GS2 and crMIR604GS2) from the immature embryo bombardment assay for editing efficiency using protoplast-mediated transfection (Table 2). AsCas12a-Ultra RNP transfection in maize protoplasts resulted in high efficiency editing at both targets, with 20.45 and 35.98% of the reads having edits, respectively. It is surprising that crMIR604GS2-RNP resulted in a high percentage of edited reads in leaf protoplasts, at even higher percentage than was observed for crBx9GS2-RNP (Table 2). It is not clear why the crMIR604GS2-RNP has a higher editing rate in leaf protoplasts as compared to immature embryos. One possible explanation is that the presence of a more open chromatin structure for the MIR604TS2 target locus in leaf *vs*. immature embryos. It should be noted that protoplast transfection has been widely applied to assess Cas9 RNP activity against different targets in various plant species including maize (Woo et al., 2015; Malnoy et al., 2016; Svitashev et al., 2016; Andersson et al., 2018; Liang et al., 2018; Lin et al., 2018; Murovec et al., 2018; Sant'Ana et al., 2020). Since a much higher percentage of cells can receive RNP in comparison with direct embryo bombardment, protoplast transfection is expected to result (on average) in much higher editing efficiencies. With our maize leaf protoplast transfection experiments with vector expressing green fluorescent protein as reporter, a typical transfection efficiency of 60–70% is obtained (results not shown). However, protoplast isolation is time-consuming and tedious, and direct bombardment of immature embryos coupled with targeted deep sequencing can offer a straightforward alternative to evaluate gRNA performance directly in the transformation target tissues.

**Table 2:** Transient editing of maize chromosomal target sequence by AsCas12a-Ultra RNP complexes delivered *via* PEG-mediated protoplast transfection.

**Experiment ID**	**Target site**	**crRNA**	**Total read number**	**Variant (InDel) read number**	**% Reads with edits (Mean ± SD)** **(rep number)**
Blank control 1	MIR604TS2	None	29,788	87	0.29% ± 0.10% (*n =* 3)
MIR604T2A	MIR604TS2	crMIR604GS2	43,716	15,729	35.98% ± 5.57% (*n =* 6)
Blank control 2	Bx9TS2	None	71,716	153	0.21% ± 0.04% (*n =* 3)
Bx9T2A	Bx9TS2	crBx9GS2	149,313	30,528	20.45% ± 15.06% (*n =* 6)

### High Efficiency Recovery of Edited Lines by Co-delivery of Cas12a RNP With a Selectable Marker Gene Vector

Since reasonable editing efficiencies were achieved using AsCas12a RNP in direct immature embryo bombardment, we also tested whether stably edited lines can be recovered from immature embryos bombarded with RNPs targeting *Bx9* gene. We co-delivered RNP with a plasmid named pBSC12672 which contains the PMI selectable marker gene (Zhong H. et al., 2018). We tested three different versions of AsCas12a nuclease: AsCas12a-WT, AsCas12a-V3 and AsCas12a-Ultra; the AsCas12a-WT and AsCas12a-V3 proteins contain the wild-type AsCas12a sequence with AsCas12a-V3 also containing an optimized NLS sequence, whereas the AsCas12a-Ultra is an engineered-version of AsCas12a-V3 with mutations that were isolated using directed evolution in bacteria to achieve higher editing efficiencies in living cells (Zhang L. et al., 2020) (Supplementary Table 1).

To optimize mutation rate, we tested multiple parameters including different versions of AsCas12a, helium pressure for bombardment and culture temperature (Supplementary Table 4). Incubation at 37°C produced more mutants but transformation efficiency was negatively affected. Overall, the results showed that 1,100 psi, 0.3 nmol RNP and culture of bombed embryos at 33°C for 2 days immediately after bombardment is a good combination for high efficiency editing of Bx9TS1 target (Supplementary Table 4). We also directly compared performance of AsCas12a-WT with AsCas12a-Ultra (Table 3). With AsCas12a-WT protein, only about 1.2–7.1% of transgenic plants derived from immature embryos co-bombarded with RNP and 12672 plasmid DNA showed successful editing at the Bx9TS1 target according to Sanger sequencing results (Table 3). The sequencing profile of 5 mutants was examined and these results indicated five distinct deletions had occurred at the expected loci (Supplementary Figure 2). Remarkably, the use of the improved AsCas12a nuclease (AsCas12a-Ultra) resulted in about a 10-fold improvement in editing rate to 68.8% (Table 3). In addition, a high percentage of the edits are biallelic, suggesting high activity of AsCas12a-Ultra in recognizing and cutting the genomic target sequences in maize cells.

**Table 3:** Targeted mutagenesis of *Bx9* target Bx9TS1 with ribonucleoprotein (RNP) complex of two versions of AsCas12a in maize co-delivered with PMI selectable marker gene vector 12672.

**Treatment**	**AsCas12a enzyme**	**Embryo explants used**	**Events** ^**#**^	**Transformation frequency**	**Putative mutant (Taqman assay)**	**Biallelic mutants**	**Seq. confirmed mutants**	**Editing rate** ^*^
A	AsCas12a-WT	2,093	84	4.0%	1	0	1	1.2%
B	AsCas12a-WT	490	99	20.2%	2	1	2	2.0%
C	AsCas12a-WT	2,165	28	1.3%	2	0	2	7.1%
D	AsCas12a-Ultra	2,670	77	2.9%	53	49	53	68.8%

# *Selectable marker plasmid (pBSC12672) was co-precipitated and co-delivered into precultured immature embryos at 1,100 psi; Bombarded embryos were incubated at 33°C for 2 days before moving back to regular culture temperature at 28°C. Calli selected with mannose*.

* *Editing rate: number of edited lines/100 regenerated plants*.

### Lines With Heritable Edits Can Be Generated Directly From Maize Immature Embryos Bombarded With Cas12a RNP Without Selection

Considering the high editing rate of AsCas12a RNP in direct immature embryo bombardment (Table 3, Supplementary Table 4), we wondered whether stably edited lines could be recovered from immature embryos that have been bombarded with RNP targeting *Bx9* gene in the absence of selection. After AsCas12a RNP was delivered into maize immature embryos, regenerated plant lines (E0) were obtained within 1 month. Putative mutants were identified using high-throughput Taqman assays (Chen et al., 2018) to identify mutations in the Bx9TS2 target sequence (Supplementary Tables 2, 5, Sequence File 1 in Supplementary Material). Mutant plants were further characterized by sequencing to confirm the identity of genomic edits. Table 4 shows that 24 mutants at Bx9TS2 target were identified from a total of 419 immature embryos bombed with AsCas12a RNP and crBx9GS2 gRNA. NGS sequencing confirmed that 16 of the 24 mutants have biallelic mutations.

**Table 4:** Mutation frequency of Bx9TS2 target in E0 plants directly generated from bombarded immature embryos with RNP without selection.

**Treatment**	**AsCas12a**	**Embryo explants used**	**Plants sampled** ^**&**^	**Biallelic mutant**	**Monoallelic mutant**	**Total number of plants with InDel**	**Editing rate** ^*^	**Editing efficiency** ^**#**^
A	V3	288	585	11	5	16	2.7%	5.6%
B	Ultra	131	204	7	1	8	3.9%	6.1%
Total		419	789	18	6	24	3.0%	5.7%

& *For this experiment only, all regenerated shoots from an explant were sampled for mutation analysis. Therefore, some explants had more than 1 shoot*.

* *Editing rate: number of edited lines/100 regenerated plants*.

# *Editing efficiency: number of edited plants/100 starting immature embryo explants*.

E0 plants were self-pollinated to produce E1 seeds. Supplementary Table 5 shows that all E1 progeny of the biallelic E0 mutants have biallelic mutations. Of the 3 monoallelic mutant E0 lines, two of them had poor germination, resulting in small number of E1 lines and thus not meaningful for statistical analysis. However, for unknown reasons line MZKE192601A571A's segregation does not fit the expected 1:2:1 (HOM/HET/WT) ratio. Overall, the results indicated that edits in E0 mutant lines generated using RNP delivery and without selection can be stably inherited to the progeny plants.

### Cas12a RNP Results in Comparable or Higher Editing Efficiency in Comparison With Editing Machinery Expressed From Transgenes

Maize transformation is usually done using particle bombardment or *Agrobacterium*-mediated delivery. Therefore, genome editing machinery is commonly delivered in the form of DNA expression cassettes. For example, Cas9 nuclease and gRNA are controlled under PolII and PolIII promoters, respectively (Svitashev et al., 2016). We were interested in comparing the relative efficiency of Cas12a editing delivered by either RNP or DNA delivery methods. For biolistic-mediated delivery of Cas12a editing machinery targeting Bx9TS1 and Bx9TS2 sites, binary vectors containing various AsCas12a and LbCas12a variants were constructed that express crRNA targeting Bx9TS1 or Bx9TS2 sequence, respectively (Supplementary Tables 2, 3, Supplementary Figure 1); Each of the vectors contains three expression cassettes: PMI selectable marker, Cas12a and crRNA flanked by self-processing ribozymes (Supplementary Figure 1). Both Cas12a and crRNA were controlled by sugarcane ubiquitin-4 promoter (prSoUbi). Embryos bombarded with different DNA vectors were then selected on mannose to recover transgenic plants. Plants were assayed for editing at Bx9TS1 and Bx9TS2 target sequences.

The results in Table 5 shows that in general the editing rate and efficiency are higher for Bx9TS2 than Bx9TS1 target sites. It is interesting that the editing efficiency of Bx9TS1 is significantly improved when higher activity mutant AsCas12a-Ultra were used in comparison with the wild-type AsCas12a protein (V3). However, the editing efficiency at Bx9TS2 was already very high with the wild-type AsCas12a protein, and the use of improved variant had a negligible effect on editing efficiency (Table 5). We also compared the editing efficiency of two different temperatures on two different editing targets Bx9TS1 and Bx9TS2. Both AsCas12a and LbCas12a vectors worked well for editing of both targets at both 28 and 33°C (Table 5). However, higher editing rates at both Bx9TS1 and Bx9TS2 target were achieved when bombed cultures were incubated at 33°C (Table 5). For LbCas12a, editing rate at both target sites is somewhat higher with LbCas12a-V3 in comparison with the LbCas12a version with rice optimized codon (Lb, Qi in Table 5).

**Table 5:** Editing efficiency of *Bx9* target sequences in transgenic plants expressing different versions of Cas12a enzymes.

**Vector ID**	**Target site**	**Cas12a gene**	**Culture temp**	**Embryo explants used**	**PMI positive events+**	**Transformation frequency**	**Biallelic**	**Monoallelic**	**Total Mutants**	**Editing rate** ^**#**^	**Editing efficiency** ^*^
pBIDT1	Bx9TS1	As, V3	33°C	714	85	11.9%	2	9	11	12.9%	1.5%
pBIDT2	Bx9TS1	As, Ultra	33°C	400	34	8.5%	9	5	14	41.0%	3.5%
pBIDT2	Bx9TS1	As, Ultra	28°C	180	12	6.7%	1	4	5	41.0%	2.8%
pBIDT3	Bx9TS1	Lb, V3	33°C	756	106	14.0%	15	7	22	23.6%	2.9%
24096	Bx9TS1	Lb, Qi	33°C	793	73	17.8%	8	7	13	17.8%	1.6%
pBIDT4	Bx9TS2	As, V3	33°C	702	132	18.8%	64	35	99	78.0%	14.1%
pBIDT5	Bx9TS2	As, Ultra	33°C	380	31	8.2%	18	10	28	83.9%	7.4%
pBIDT5	Bx9TS2	As, Ultra	28°C	205	15	7.3%	3	3	6	33.3%	2.9%
pBIDT6	Bx9TS2	Lb, V3	33°C	720	74	10.3%	30	30	60	83.8%	8.3%
24100	Bx9TS2	Lb, Qi	33°C	787	86	10.9%	30	26	58	67.4%	7.4%

# *Editing rate: number of edited lines/100 transgenic events*.

* *Editing efficiency: number of edited plants/100 starting immature embryo explants. For 33°C treatment, bombed embryos were incubated at 33°C for 2 days before moving back to regular culture temperature at 28°C*.

It is also interesting that the editing rate of Bx9TS1 target sequence with AsCas12a-Ultra RNP was higher than when the editing machinery was expressed from a transgene (Tables 3, 5). The lower editing efficiency with AsCas12a expressed from a transgene is probably due to several reasons. While DNA delivery is efficient, efficient transcription and translation of delivered transgenes can be highly variable depending on the activity of promoters and post-transcriptional regulatory features, and poor codon optimization can also negatively influence overall transgene expression. DNA transgene expression also depends on proper gRNA processing and transport of Cas12a protein back to the correct nuclear compartment for proper RNP assembly; whereas *in vitro* assembly of Cas12a RNP is very efficient and the pre-assembled RNP can start doing editing once delivered into the nucleus.

## Conclusion

CRISPR-Cas12a is an attractive alternative system to CRISPR-Cas9 for crop genome editing (Zetsche et al., 2015; Tang et al., 2017). However, it has several limitations in comparison with Cas9 including less flexibility due to its longer PAM sequence requirements (TTTV-3′), low activity at room temperature, higher percentage of non-functional gRNA (Lee et al., 2019, Malzahn et al., 2019). Therefore, it is highly desirable to have a fast and efficient prescreen system for gRNAs or Cas12a expression vectors to test their functionality before investing resources to carry out stable transformation which is resource-intensive and also usually takes 2–3 months before regenerated materials are available for molecular analysis. In this report we have demonstrated that the commonly used maize transformation target explants, i.e., immature embryos can be directly bombed with RNPs for assessing their genome editing capability. Alternatively, leaf protoplasts can be an efficient RNP screening system. We have also shown that RNP led to high efficiency editing when delivered into maize immature embryo targets; In addition, stable lines with heritable edits can be efficiently generated *via* RNP delivery with or without co-selection. Finally, we have demonstrated that both AsCas12a, LbCas12a and their mutants with enhanced activities can be used to generate targeted genome modifications at high rate. The techniques described in this paper provide us useful tools for precision genome engineering of maize, an important field crop.

## Data Availability Statement

The datasets used and/or analyzed during the current study are available from the corresponding author on reasonable request.

## Author Contributions

SD, WG, MY, SR, and QQ conceived and designed experiments. YQ, CV, and MC constructed the vectors. AM, SE, MM, and SD did transformation experiments. SR did protoplast experiments. CF, YJ, LZ, MY, and WG did assay design and molecular analysis. CV and MC provided Cas12a enzymes. QQ, HZ, ZC, and LS provided suggestions, research, and laboratory support. SD and QQ wrote the manuscript. All authors contributed to the article and approved the submitted version.

## Conflict of Interest

SD, YQ, MM, SR, LZ, YJ, WG, CF, AM, ZC, MY, HZ, SE, LS, and QQ were employed by Syngenta Crop Protection, LLC. CV and MC are employed by Integrated DNA Technologies which manufactures genome editing reagents.
