# Supplementary material for: Efficient Targeted Mutagenesis Mediated by CRISPR-Cas12a Ribonucleoprotein Complexes in Maize
Source: Front Genome Ed. 2021 May 12;3:670529. doi: 10.3389/fgeed.2021.670529 (PMC8525364; doi:10.3389/fgeed.2021.670529)
Supplement: Supplementary file 1 [file Data_Sheet_1.zip › Suppl. Datafile 1.DOCX]

**Supplementary Sequence File 1**. Wild type NP2222 maize *Bx9* sequence (cZmUGTBx9-02, 1,488 bp) (Note there is a small intron, underlined and highlighted in yellow, cRNA target sequences of Cas12a vectors (24096/Bx9TS1 and 24100/Bx9TS2) including PAM are highlighted in purple)

atggcgtcgtcgcgcaccggagccggagccggagccggcggccgtgtggtggtcttcccgttcccgttccagggccacttcaacccggtgatgcggctggcccgcgcgctgcacgcccggggcctcgcgattaccgtcttccacagcggcgccctggacccggccgactaccccgccgactaccgcttcgtgcccgtgaccgtggaggcggacccgaagctgctggcgtccgaggacatcgccgccatcgtcaccacgctgaacgccagctgcgatgcccccttcagggcccgcctctcggcgctgctggccgccgaggggagggacagcgtccggtgcgtcttcaccgacgtcagctggaacgccgtgctgacggcgtccagcgacctcggcgtgcccgcgctcggcatgatgacggccagcgccgcctcgttacgcgactacatggcgtaccgcaccttgatcgacaagggctacctgccggtgaaaggtgagtccatctccgtctccatccaatcgtcgtcggcgaggttattagcagagggagactagattagatttgcttatcatcacatacctgcagaggagcgcaaggaggatcccgtacccgagctacccccgtaccgcgtcaaagacctgctccgggtcgacacgtccgacctggaggagttcgccgaactgctggcccgcaccgtcaccgcggcgcggcgcgcctcggggctcatcttcaacaccttcccgctgatcgagacagacacgctggccgagatccacaaggccttgtcggtgccggtgttcgccgtcgccccgctcaacaagctggtgccgacggccacggccagcctgcacggggtggtccaggcggaccggggctgcctgcagtggctggacacgcagcagccgggctccgtgctgtacgtcagcttcgggagcatggccgccatggacccgcacgagttcgtggagctcgcgtgggggctcgccgacagcaagcgccccttcgtgtgggtggtcaggcccaatctcatccgcggcttcgagtccggcgcgctgcccgacggggtggaggacgaggtgcgcggccgcggcatcgtcgtcacctgggcgccgcaggaggaggtgctcgcgcacccggccgtcggcggcttcctcacccacaacggttggaactccaccgtcgaggccatctcggagggcgtgcccatggtctgctgcccgcggcacggggaccagttcggcaacatgaggtacgtgtgcgacgtgtggaaggtgggcacggagctcgtgggggaacagctggagagaggccaggtcaaggccgccatcgacaggctctttggcaccaaggaaggggaggagatcaaggagaggatgaaggaattcaagatcgctgcggccaaaggcatcggcatcggcgtcgacgtcgacgaaactacgtcaccccgcacggatttaaccgatttggttgatctcataaaatccttctga
