# Supplementary material for: Efficient Targeted Mutagenesis Mediated by CRISPR-Cas12a Ribonucleoprotein Complexes in Maize
Source: Front Genome Ed. 2021 May 12;3:670529. doi: 10.3389/fgeed.2021.670529 (PMC8525364; doi:10.3389/fgeed.2021.670529)
Supplement: Supplementary file 1 [file Data_Sheet_1.zip › Suppl. Datafile 2.DOCX]

**Supplementary Sequence File 2**. NP2222 maize MIR604FS sequence (bp) (Note: cRNA target sequences MIR604TS1, MIR604TS2, MIR604TS3 and MIR604TS4 (Suppl. Table 2) including PAM are highlighted in purple)

ACGGACGAGGTGTCGTGAGAACCGGCAAAAAAAAAAATCATCGCAAGTGCGCTGAAGTGAAGTGCCTTCCCCCGCGTTTCCTTGCCCCTGGCCGGTACCCATTTGGCGCCGATTCTTTTCTTGCCCCCCGGCCGGCCGCTCGCTCGCCTTTGGATTCTTCCAAAGCCGCTGATGGGATGGTGGCGAACACACCCACCACCCGTCTTTGCCCAAAGCGACCCGGCACAGGCCGCGCCGGCTTCACTAACCACTAGCGCTTGTACTAATAAAATGGTTTCTAGCGTTTGTTGCTCTCCTTTTTCTTTTTTCGCCGGTTCTTCGGAGCCGTGTGGACACTGGACAGCGTCCAGTCCAGCAGGCATAGGGTGGTCTCGGCGGCGGTCGTCCGACGACGATCGATCTCCATGAGATTCCGCGACAGGCCAGGACGGAAAGCTGGGCCCTTCTCACCAATTCGCGTCGGAGCCGGAACAAGATTCCCTCCCCCAATCATTTCGACGCGCCCTTTCTTCGCCACCCCTCGTGGCCGTGTTTCGCGGCCGGCCCTTATCTCCTTCCCGTGACGCGTTCTTTTGTAGCTTAGCGGCCGGCACGTTGCTAACCAGGCTAGCTTCGTTCGTTTTTAATCTGCCTATCGAGAAGAGAAGAAAAATTCGTCCATGGGGCCACGGCCTCTTCTGCAGGCATTTGGCATGTGAAGGAACCCGAACCAGTGAATGGAGATGGACGGATGCTGCTCAGATACGCAGTCAAACCTGCCGGCGAAATTACGGGGGGAGCTGGCTGGCTGGCTGGCTGGACGCCAGATCACACATGGATGACGCGGCACGGCAGCTAGCCGAGCAGGCGCTCTGCGCACGCAAGTGTCGTGCCGATCTCGCACCAGCAGCATCGCGTCCTAAACAAAGGAGGTCCTGTCCTGCACTGCACTGCACTGCACGGATGCAGCTTTGGCAACGAGGTGTGTCGCGCAGCGCTCCTGCACGGATGTAGCTTTGGATTGCTGGATAATGTCTCGCGCAAGCGTCGTATTTATTTATTTATTTATTACAGCCTCCACCGCCGTGCGTGCTCCGTTTCGGATTA
