# Supplementary material for: Efficient Targeted Mutagenesis Mediated by CRISPR-Cas12a Ribonucleoprotein Complexes in Maize
Source: Front Genome Ed. 2021 May 12;3:670529. doi: 10.3389/fgeed.2021.670529 (PMC8525364; doi:10.3389/fgeed.2021.670529)

**Supplementary Figure 2**. Sanger sequencing confirmation of mutant plants produced via delivery AsCas12a- gRNA RNP targeting Bx9TS1 sequence


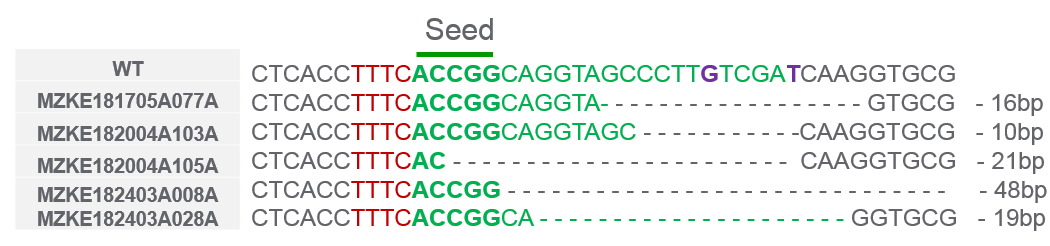

Supplement: Supplementary file 1 [file Data_Sheet_1.zip › Suppl. Figure 2.DOCX]
