# Supplementary material for: Efficient Targeted Mutagenesis Mediated by CRISPR-Cas12a Ribonucleoprotein Complexes in Maize
Source: Front Genome Ed. 2021 May 12;3:670529. doi: 10.3389/fgeed.2021.670529 (PMC8525364; doi:10.3389/fgeed.2021.670529)

**Supplemental Figure 3.** NGS sequencing results of Bx9TS2 target in the edited E0 lines from Table 4 (16 of the 24 mutants have biallelic mutations)


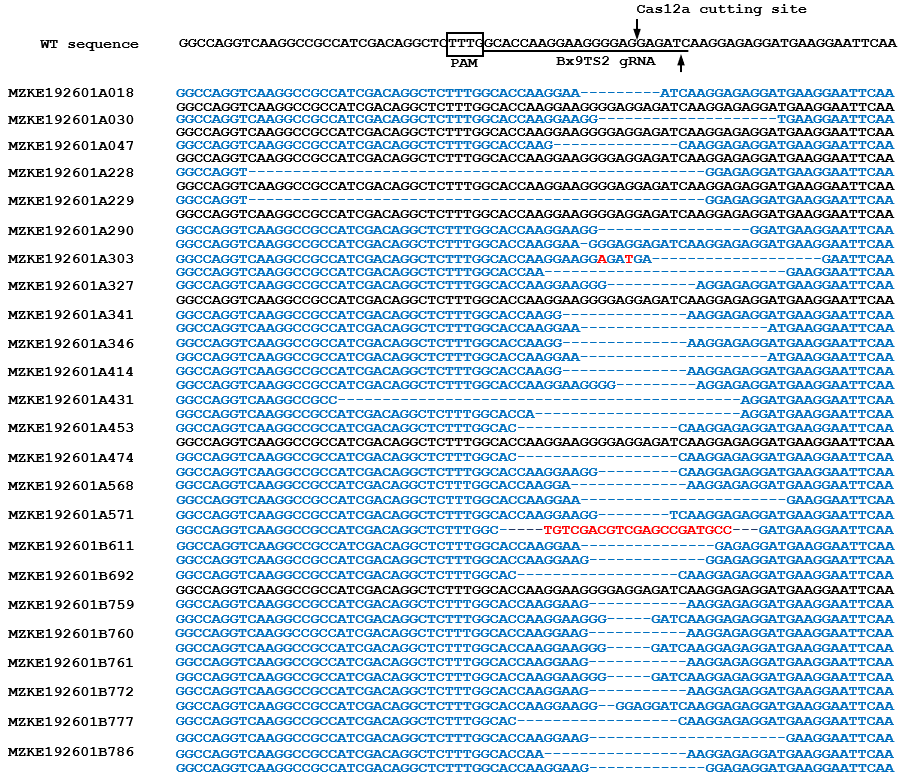

Supplement: Supplementary file 1 [file Data_Sheet_1.zip › Suppl. Figure 3.DOCX]
