# Supplementary material for: Efficient Targeted Mutagenesis Mediated by CRISPR-Cas12a Ribonucleoprotein Complexes in Maize
Source: Front Genome Ed. 2021 May 12;3:670529. doi: 10.3389/fgeed.2021.670529 (PMC8525364; doi:10.3389/fgeed.2021.670529)
Supplement: Supplementary file 1 [file Data_Sheet_1.zip › Suppl. Table 1.DOCX]

**Supplementary Table 1.** Cas12a/Cpf1 enzymes from IDT used in this study

| Cas12a enzyme and version | Cas12a protein sequence | Linker | NLS |
| --- | --- | --- | --- |
| AsCpf1-WT  (Behlke et al., 2018) | Wild type | N/A | Original (SV40) |
| Alt-R® AsCas12a (Cpf1) V3 (Zhang et al., 2020) | Wild type | Improved (4xGGSGGS) | Optimized  (OpT) |
| Alt-R® AsCas12a (Cpf1) Ultra (Zhang et al., 2020) | Optimized mutant (M537R, F870L,) | Improved | Optimized  (OpT) |
| LbCpf1-V3  (Behlke et al., 2018) | Wild type | Improved | Optimized  (OpT) |
