# Supplementary material for: Efficient Targeted Mutagenesis Mediated by CRISPR-Cas12a Ribonucleoprotein Complexes in Maize
Source: Front Genome Ed. 2021 May 12;3:670529. doi: 10.3389/fgeed.2021.670529 (PMC8525364; doi:10.3389/fgeed.2021.670529)
Supplement: Supplementary file 1 [file Data_Sheet_1.zip › Suppl. Table 2.DOCX]

**Supplementary Table 2.** gRNA sequences for RNP delivery

| Target gene | crRNA ID | Spacer/gRNA sequence | Target region |
| --- | --- | --- | --- |
| Bx9TS1 (aka ZmBx9Target3r) | crBx9GS1 | 5’-ACCGG CAGGT AGCCC TTGTC GAT -3’ | Genic, upstream of the intron |
| Bx9TS2 (aka ZmBx9Target2) | crBx9GS2 | 5’-gcacc aagga agggg aggag atc -3’ | Genic, protein coding sequence |
| MIR604TS1 | crMIR604GS1 | 5’-gacgc gccct ttctt cgcca ccc -3’ | intergenic |
| MIR604TS2 | crMIR604GS2 | 5’-Gcatg tgaag gaacc cgaac ca -3’ | intergenic |
| MIR604TS3 | crMIR604GS3 | 5’-gattg ctgga taatg tctcg cgc -3’) | intergenic |
| MIR604TS4 | crMIR604GS4 | 5’-cccaa agcga cccgg cacag gcc -3’ | intergenic |
