# Supplementary material for: Efficient Targeted Mutagenesis Mediated by CRISPR-Cas12a Ribonucleoprotein Complexes in Maize
Source: Front Genome Ed. 2021 May 12;3:670529. doi: 10.3389/fgeed.2021.670529 (PMC8525364; doi:10.3389/fgeed.2021.670529)
Supplement: Supplementary file 1 [file Data_Sheet_1.zip › Suppl. Table 3.DOCX]

**Supplementary Table 3** CRISPR-Cas12a vectors and their target DNA sequences in the maize *Bx9* gene (*PAM sequences for LbCas12a nuclease target recognition). Schematic drawing of the vector is shown in Suppl. Figure 1 for 24096 and 24100.

| Vector ID. | Bx9 target sequence | Cas12a gene, version |
| --- | --- | --- |
| pBIDT1 | Bx9TS1 | AsCas12a, V3 (Suppl Table 1) |
| pBIDT2 | Bx9TS1 | AsCas12a, Ultra (Suppl Table 1) |
| pBIDT3 | Bx9TS1 | LbCas12a, V3 (Suppl Table 1) |
| 24096 | Bx9TS1 | LbCas12, Qi lab (Tang et al., 2017) |
| pBIDT4 | Bx9TS2 | AsCas12a, V3 (Suppl Table 1) |
| pBIDT5 | Bx9TS2 | AsCas12a, Ultra (Suppl Table 1) |
| pBIDT6 | Bx9TS2 | LbCas12a, V3 (Suppl Table 1) |
| 24100 | Bx9TS2 | LbCas12, Qi lab (Tang et al., 2017) |
