# Supplementary material for: Efficient Targeted Mutagenesis Mediated by CRISPR-Cas12a Ribonucleoprotein Complexes in Maize
Source: Front Genome Ed. 2021 May 12;3:670529. doi: 10.3389/fgeed.2021.670529 (PMC8525364; doi:10.3389/fgeed.2021.670529)
Supplement: Supplementary file 1 [file Data_Sheet_1.zip › Suppl. Table 4.DOCX]

**Supplementary Table 4.** Testing multiple parameters for improved editing rate at Bx9TS1 target site

| **Treatment** | **Alt-R® AsCas12a** | **Psi** | **Incubation temp (**^o^C**)** | **Explants** | **Regenerated plants** | **PMI+ plants** | **TF%** | **Mutants** | **editing rate*** | **Editing efficiency#** |
| --- | --- | --- | --- | --- | --- | --- | --- | --- | --- | --- |
| A | V3 | 1100 | 33 | 345 | 68 | 57 | 16.50% | 36 | 63.20% | 10.4% |
| B | V3 | 1100 | 37 | 228 | 23 | 22 | 9.60% | 9 | 40.90% | 3.9% |
| C | V3 | 1100 | 33 | 361 | 57 | 52 | 14.40% | 25 | 48.10% | 6.9% |
| D | Ultra | 1350 | 33 | 352 | 18 | 17 | 4.80% | 10 | 58.80% | 2.8% |
| E | Ultra | 1100 | 37 | 401 | 29 | 27 | 6.70% | 22 | 81.50% | 5.5% |

Note: Selectable marker plasmid (pBSC12672) was co-precipitated and co-delivered in this set of experiments; 0.3 nmol each of Cas12a enzyme and crRNA were used for RNP per shot. *Editing rate: edited line/100 transgenic events; #Editing efficiency: edited plants/100 embryo explants
