# Supplementary material for: Efficient Targeted Mutagenesis Mediated by CRISPR-Cas12a Ribonucleoprotein Complexes in Maize
Source: Front Genome Ed. 2021 May 12;3:670529. doi: 10.3389/fgeed.2021.670529 (PMC8525364; doi:10.3389/fgeed.2021.670529)
Supplement: Supplementary file 1 [file Data_Sheet_1.zip › Suppl. Table 5.DOCX]

**Supplementary Table 5** Mutations in E0 plants are heritable and passed to the E1 progeny

|  | E0 | E1 seed germination | | | Number of E1 plants* | | |
| --- | --- | --- | --- | --- | --- | --- | --- |
| Mutant ID | Mutation type* | Seed Quantity | Number of seeds planted | Seeds germinated (Plants genotyped) | WT | Monoallelic mutant | Biallelic mutant |
| MZKE192601A760A | monoallelic | 287 | 128 | 17 | 0 | 8 | 9 |
| MZKE192601A414A | biallelic | 336 | 64 | 36 | 0 | 0 | 36 |
| MZKE192601A571A | monoallelic | 201 | 128 | 108 | 27 | 81 | 0 |
| MZKE192601A759A | monoallelic | 284 | 128 | 29 | 0 | 11 | 18 |
| MZKE192601A346A | biallelic | 99 | 64 | 47 | 0 | 0 | 47 |
| MZKE192601A030A | biallelic | 271 | 64 | 51 | 0 | 0 | 51 |
| MZKE192601A018A | biallelic | 363 | 64 | 50 | 0 | 0 | 50 |
| MZKE192601A611A | biallelic | 204 | 64 | 43 | 0 | 0 | 43 |
| MZKE192601A786A | biallelic | 150 | 64 | 49 | 0 | 0 | 49 |

*Mutation type is based on target sequence copy number call: 0 copy represent biallelic mutation, 1 copy represent monoallelic mutation, 2 copy represent wild type sequence detected
